# Sodium-glucose Co-transporter-2 Inhibitors Causing *Candida tropicalis* Fungemia and Renal Abscess

**DOI:** 10.1210/jcemcr/luae010

**Published:** 2024-02-01

**Authors:** Prathap Kumar Simhadri, Pradeep Vaitla, Sriram Sriperumbuduri, Deepak Chandramohan, Prabhat Singh, Ujjwala Murari

**Affiliations:** Division of Nephrology, Advent Health/FSU School of Medicine, Daytona Beach, 32117 FL, USA; Division of Nephrology, University of Mississippi Medical Center, Jackson, 39216 MS, USA; Division of Nephrology, University of Mississippi Medical Center, Jackson, 39216 MS, USA; Division of Nephrology, University of Alabama School of Medicine, Birmingham, 35233 AL, USA; Division of Nephrology, Christus Spohn Hospital, Corpus Christi, 78404 TX, USA; Division of Nephrology, West Virginia University, Morgantown, 26506 WV, USA

**Keywords:** SGLT2I, fungemia, fungal infection, drug-induced fungemia, sodium-glucose cotransporter-2 inhibitor

## Abstract

Sodium-glucose cotransporter-2 inhibitors (SGLT2i) are a relatively newer class of medications, approved by the U.S. Food and Drug Administration in 2013 to treat type 2 diabetes mellitus. Over the past few years, the indications for SGLT2i have been expanded to decrease the risk of kidney disease and cardiovascular disease. SGLT2i are associated with an increased risk of euglycemic diabetic ketoacidosis, urinary tract infections, and genital mycotic infections. There are a few case reports of severe invasive fungal infections due to Candida in patients using SGLT2i. We present the case of *Candida tropicalis* fungemia and renal abscess in a patient on an SGLT2i.

## Introduction

Sodium-glucose cotransporter-2 inhibitors (SGLT2i) are a relatively newer class of medications approved by the Food and Drug Administration in 2013 to treat type 2 diabetes mellitus [1]. Over the past few years, the indications for SGLT2i have been expanded to decrease the risk of kidney disease and cardiovascular disease [2]. SGLT2i blocks glucose reabsorption in the proximal convoluted tubule of the nephron, thereby increasing urinary glucose excretion and decreasing blood glucose concentration [3]. Downstream effects of increased glucose elimination include natriuresis leading to decreased plasma volume, reduced vascular resistance, and lowered systemic blood pressure [4].

Despite the broad benefits of SGLT2i, several adverse events have been reported, including euglycemic diabetic ketoacidosis, urinary tract infections (UTIs), genital mycotic infections with all the agents, and a risk of lower limb amputation with canagliflozin specifically [5]. A handful of case reports have described the occurrence of severe invasive fungal infections due to Candida in patients on SGLT2i [6‐9]. We describe a case of invasive Candida infection in a patient on empagliflozin following a prostate biopsy.

## Case Presentation

A 75-year-old Caucasian male with a history of type 2 diabetes mellitus for more than 10 years, hypertension, obesity, chronic kidney disease stage IIIB with baseline creatinine of 1.6 mg/dL (141.44 µmol/L) (normal reference range: 0.5-1 mg/dL or 74.3-107 µmol/L), and benign prostatic hyperplasia presented to the hospital with a 1-day history of fever and lethargy, 1 week after the transrectal prostate biopsy. Home medications included valsartan 320 mg daily, glimepiride 4 mg daily, metformin 500 mg twice daily, and empagliflozin 25 mg daily. He was previously on canagliflozin 300 mg daily for more than 2 years, and it was changed to empagliflozin 25 mg daily about 9 months prior to presentation.

Initial vital signs showed a T-max of 102.8° F, a heart rate of 92 beats per minute, a blood pressure of 106/50 mm of Hg, and a respiratory rate of 17 breaths per minute. The patient was lethargic and confused and exhibited signs of encephalopathy. Examination of other systems was nonrevealing.

## Diagnostic Assessment

Laboratory evaluation showed significant leukocytosis with bandemia, with a rise in serum creatinine from his baseline. He also had lactic acidosis and elevated procalcitonin levels. The detailed results of his blood count and other pertinent labs are shown in Table 1.

**Table 1.** The labs with reference ranges

Lab	Results	Normal range
WBC	**25 100/mm^3^ (25.1 × 10^9^/L)**	4500-11,000/mm^3^ (4.5-11.0 × 10^9^/L)
Bands	**21% (21)**	0-3% (0-3)
Hemoglobin	**11.2 g/dL (6.95 mmol/L)**	13.5-17.5 g/dL (8.38-10.86 mmol/L)
Hematocrit	**34% (34)**	41-53% (41-53)
Platelet count	187 000/mm^3^ (187 × 10^9^/L)	150,000-400,000/mm^3^ (150-400 × 10^9^/L)
BUN	**44 mg/dL (15.7 mmol/L)**	8-25 mg/dL (2.9-8.9 mmol/L)
Creatinine	**3.56 mg/dL (314.71 µmol/L)**	0.5-1 mg/dL (74.3-107 µmol/L)
Sodium	**134 mmol/L (134 mmol/L)**	135-145 mmol/L (135-145 mmol/L)
Potassium	5.0 mmol/L (5.0 mmol/L)	3.4-5.0 mmol/L (3.4-5.0 mmol/L)
Bicarbonate	**18 mmol/L (18 mmol/L)**	20-32 mmol/L (20-32 mmol/L)
Procalcitonin	**46.93 ng/mL (46.93 µg/L)**	0.05 ng/mL (0.05 µg/L)
Lactic acid	**2.9 mmol (2.9 mmol/L)**	0.5-2.2 mmol/L (0.5-2.2 mmol/L)
Beta-hydroxybutyrate	0.27 mmol/L (0.27 mmol/L)	0.4-0.5 mmol/L (0.4-0.5 mmol/L)
Hemoglobin A1C	**7.9%**	<5.7%
Blood glucose	**289 mg/dL (16.04 mmol/L)**	70-110 mg/dL (3.9-6.1 mmol/L)

Abbreviations: BUN, blood urea nitrogen; WBC, white blood cells.

Abnormal values are shown in bold font. Values in parenthesis are international system of units.

The urine dipstick showed 3+ glucose, 3+ blood, 2+ protein, 3+ leukocyte esterase, negative nitrite, and trace ketones. Urine microscopy showed >60 white blood cells per high power field (HPF), (normal reference range: ≤2-5 white blood cells), >30 red blood cells per HPF (normal reference range: ≤2 red blood cells), and 3+ yeast per HPF (normal reference range: none).

**Figure 1.**
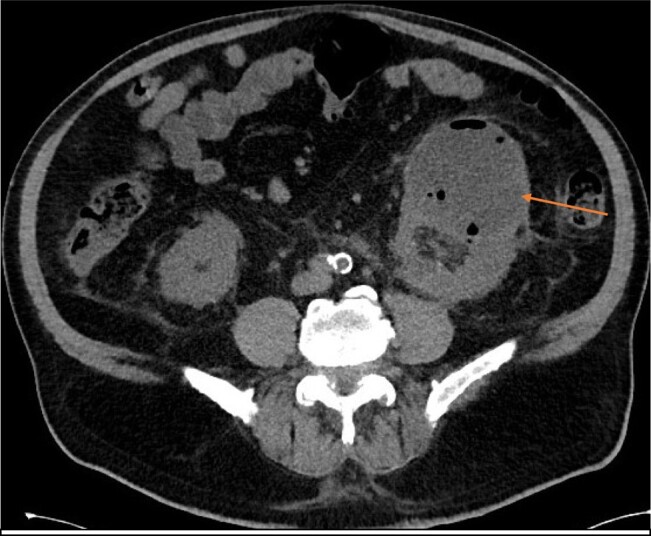
The noncontrast image of the computed tomography abdomen showing a left renal abscess with fluid and air (marked with an arrow).

A computerized tomography scan of the abdomen and pelvis without intravenous (IV) contrast was done given his acute kidney injury, and it showed fluid in the superior pole of the left kidney with surrounding inflammatory changes concerning renal abscess, measuring 7.6 × 8.0 × 8.9 cm, as shown in Fig. 1. There were inflammatory changes in the left renal collecting system with thickening of the urothelium and bladder wall.

## Treatment

The patient was diagnosed with septic shock and metabolic encephalopathy. Blood and urine cultures were sent; he was resuscitated with IV fluids; and he was started on broad-spectrum antibiotics, IV piperacillin/tazobactam, and vancomycin therapy.

He underwent percutaneous drainage of the renal abscess under computed tomography guidance. Blood and urine cultures and abscess fluid showed *Candida tropicalis* on day 2 of hospitalization.

Infectious diseases consultation was obtained, and he was started on IV micafungin 150 mg daily on hospital day 2 based on their recommendations. He was started on renally adjusted IV fluconazole 200 mg daily in place of micafungin on day 3 of the hospitalization as the *Candida tropicalis* was pan-sensitive to caspofungin, fluconazole, micafungin, and voriconazole. The dose was later adjusted to 400 mg daily once his renal function had improved, and it was later transitioned to oral fluconazole therapy on day 9 at 400 mg daily for a total of 6 weeks. He was discharged home in stable condition after a few days, and an SGLT2i was withheld from the patient.

## Outcome and Follow-up

He was continued on metformin and glimepride therapy, and the sitagliptin was later added for his diabetes management; the SGLT2i was never resumed. He continued to have recurrent hospitalizations secondary to fungal UTIs and recurrent renal abscesses. He later underwent a partial left nephrectomy.

## Discussion

SGLT2i are being widely prescribed due to their beneficial effects on kidney and cardiovascular outcomes among the diabetic and nondiabetic populations [10]. A recent meta-analysis of 13 trials has shown relative risk reduction in the risk of kidney disease progression by 37%, risk of acute kidney injury by 23%, and risk of cardiovascular death or hospitalization of heart failure by 23%, with similar effects in patients with and without diabetes [10]. This study also highlighted the complications associated with SGLT2i, including the risk of UTIs (relative risk 1.08, 95% confidence interval 1.02-1.15) and mycotic genital infections (relative risk 3.57, 95% confidence interval 3.14- 4.06) [10].

Glycosuria secondary to inhibition of the SGLT2 channel in the proximal tubular epithelial cells by these drugs provides a favorable substrate for the growth of microorganisms [11]. Among people with diabetes, the risk is further increased by the impaired immune system, poor metabolic control, and incomplete bladder emptying due to autonomic neuropathy [12]. A retrospective cohort study of 584 patients identified lower baseline estimated glomerular filtration rate and history of genitourinary infection as predictors for a higher risk of genitourinary infection after initiating SGLT2i [13]. Another study comparing patients initiated on SGLT2i and dipeptidyl peptidase 4 inhibitors showed a higher prevalence of genital infections with SGLT2i (within 1 year, 8.1% with SGLT2i vs 1.8% with dipeptidyl peptidase 4 inhibitors), and the key predictors of the infection were female sex and history of prior infection [14].

There were very few case reports of fungemia described among patients on SGLT2i. Table 2 summarizes the reported cases of fungemia associated with SGLT2i therapy.

**Table 2.** The reported fungemia cases so far with SGLT2i

Case number	Demographics	Risk factor(s)	SGLT2i used	Organism(s) isolated from blood and urine	Treatment and outcome
1 [6]	64-year-old-male	T2DM, obstructive uropathy secondary to nephrolithiasis	Empagliflozin for 2 years	Candida albicans in blood, urine, and urethral swab cultures	IV anidulafungin, then 6 weeks of fluconazole. Right-sided emphysematous pyelonephritis with obstructing right renal calculus s/p right nephrostomy tube and ureteral stent and emphysematous prostatitis and abscess s/p transurethral drainage and then resection of the prostate. Patient recovered
2 [6]	72-year-old male	T2DM, urethral stricture	Empagliflozin for 2 months	Candida glabrata in blood, urine, and urethral swab cultures	IV caspofungin, then IV micafungin, fluconazole, and 5-flucytosine for 4 weeks, s/p urethral dilatation
3 [7]	76-year-old male	T2DM, ureterostomy following cystectomy for urothelial carcinoma	Drug not specified	Candida glabrata in blood cultures	IV anidulafungin
4 [8]	38-year-old female	T2DM, cystoscopy and double-J ureteral stent for left distal ureteric stone with mild hydroureteronephrosis	Canagliflozin for 14 months	Candida glabrata in blood and urine and Klebsiella pneumonia in urine	Oral fluconazole for 2 weeks+ cefazolin for 7 days Left ureteral stent exchanged. Patient improved
5 [9]	67-year-old-female	T2DM for 11 months, ureteral stone causing right moderate hydronephrosis	Empagliflozin for 9 months	Candida glabrata and ESBL E coli in blood and urine cultures	IV ertapenem and micafungin for 2 weeks. Ureteral stones s/p right ureteral stenting, then percutaneous nephrostomy. Fungal ball in the renal pelvis s/p amphotericin B irrigation via the nephrostomy tube and ureteroscopic removal
6 (current patient)	75-year-old- male	T2DM, prostate biopsy	Empagliflozin for 9 months and canagliflozin for 2 years prior to that	Candida tropicalis in blood and urine culture	IV micafungin, then IV and oral fluconazole for 6 weeks. Left lower pole renal abscess drainage

Abbreviations: ESBL E coli, extended-spectrum beta-lactamase Escherichia coli; IV, intravenous; SGLT2i, sodium-glucose co-transporter inhibitor; s/p, supported by; T2DM, type 2 diabetes mellitus.

Candida species account for most fungal UTIs and usually present as complicated nosocomial infections. Positive urine cultures for fungi rarely occur in a community setting with a clean-catch specimen, whereas the incidence increases significantly among hospitalized patients with indwelling catheters [15]. Risk factors for fungal UTI include diabetes mellitus, use of broad-spectrum antibiotics, indwelling urinary catheters, female sex, extremes of age, exposure to immunosuppressive agents, and poor urinary flow. Diabetes mellitus promotes colonization of the genital tract due to impaired phagocytic activity and can cause neurogenic bladder, leading to urinary stasis. There is a higher incidence of vaginitis and balanitis among diabetic women and men compared to nondiabetics.

SGLT2i, through the induction of renal glycosuria, creates a conducive environment for the proliferation and colonization of Candida in the genital tract. This effect is exacerbated in patients with obstructive uropathy resulting from conditions such as stones or strictures and those with poor urine flow due to an enlarged prostate or neurogenic bladder. In such cases, instrumentation of the genital tract (as in our patient who underwent a prostate biopsy) or breach of the urothelium can lead to the dissemination of the Candida fungus into the bloodstream, causing systemic sepsis.

Our case highlights the risk of fungemia in patients taking SGLT2i and undergoing urological procedures, including a prostate biopsy. Our patient was not exposed to prolonged antibiotics and had no other known predisposing factor that could contribute to fungemia.

## Learning Points

- Clinicians should recognize an increased risk of fungemia and invasive fungal infections with SGLT2i in the specific patient population.
- Patients with complex urinary anatomy, severe benign prostatic hyperplasia, or any other condition causing urinary outlet obstruction would be at high risk of developing invasive fungal infections. The risks and benefits of administering SGLT2i in this population should be thoroughly evaluated.
- SGLT2i therapy should be held temporarily in patients undergoing elective urological interventions.
- Antifungal prophylaxis should be considered in SGLT2i therapy patients requiring urgent invasive urological procedures.

## Contributors

All authors made individual contributions to authorship. P.K.S. was involved in diagnosing and managing this patient. P.K.S., P.V., and S.S. prepared a preliminary manuscript. D.C., P.S., and U.M. prepared the final draft of the manuscript. All authors reviewed and approved the final draft.

## Data Availability

Data sharing is not applicable as no data sets were generated or analyzed during the current study.
